# OK-432 Administration Inhibits Murine Allergic Rhinitis at the Induction Phase, through the Macrophage Activation with TLR2 Signaling Pathway

**DOI:** 10.3390/medsci6040107

**Published:** 2018-11-26

**Authors:** Noriaki Aoi, Ichiro Morikura, Takafumi Fuchiwaki, Takaya Yamada, Emmanuel Prokopakis, Hideyuki Kawauchi

**Affiliations:** 1 Department of Otorhinolaryngology, School of Medicine, Shimane University, 89-1 Enya-cho, Izumo, Shimane Prefecture 693-8501, Japan; nori-aoi@med.shimane-u.ac.jp (N.A.); i-moriku@med.shimane-u.ac.jp (I.M.); fuchi@med.shimane-u.ac.jp (T.F.); 2 Department of Experimental Animals Center for Integrated Research in Science, Shimane University, 89-1 Enya-cho, Izumo, Shimane Prefecture 693-8501, Japan; yamada-t@med.shimane-u.ac.jp; 3 Department of Otorhinolaryngology-Head and Neck Surgery, University Hospital of Crete, 711 10 Heraklion, Greece; emmanuel@prokopakis.gr

**Keywords:** OK-432, TLR2, allergic rhinitis, macrophage

## Abstract

OK-432, a preparation of a low-virulence strain (Su) of *Streptococcus pyogenes* (Group A) killed by a penicillin and lyophilized, is a stiff inducer of Th1 cytokines, and exerts anti-cancer effects in tumor-bearing mice. OK-432 has been reported to consist of many bacterial components, such as peptidoglycan, M-protein, etc. However, it is yet to be ascertained which bacterial component induces T helper 1 (Th1) responses. For the last decade, Toll-like receptor (TLR) family proteins are well elucidated to play a role in recognizing bacterial components and inducing interleukin (IL)-12 from macrophages. Above all, peptidoglycan seems to be the agonist of TLR2 rather than the obverse. In our present study, the role of TLR2 for the recognition of OK-432 by macrophages and the effects of OK-432 are examined on murine allergic rhinitis model. Interestingly, results show IL-12 production by macrophages derived from TLR2 knock-out (ko) mice was significantly decreased, in comparison with that of macrophages derived from wild-type mice. Moreover, in TLR2 ko mice, no regulatory effect of OK-432 was observed on an allergic rhinitis model. These data indicate that TLR2 signaling is involved in regulating OK-432-induced anti-T helper 2 (Th2) immunity, and may offer a new prophylactic and therapeutic approach using OK-432 to downregulate allergic disorders, such as allergic rhinitis.

## 1. Introduction

The exact pathology of allergic rhinitis is well known to be an immediate type hypersensitivity connected with an expansion of T helper 2 (Th2) responses, including the production of allergen-specific immunoglobulin (Ig) E and nasal cavity infiltration of eosinophils [1,2,3,4,5]. Allergic reaction can be allocated to two phases: systemic response at the induction phase and local allergic inflammation at the eliciting phase. Naive clusters of differentiation (CD) 4^+^ T cells initially stimulated with an allergen in the presence of interleukin (IL)-4 actually develop into CD4^+^ T cells, which secrete IL-4, IL-5, IL-6 and IL-13 for IgE isotype switching [6,7,8]. In case the same allergen is inhaled, the allergen inevitably crosslinks preformed IgE bound to high-affinity Fcε_receptor (FcεR) on mast cells residing in nasal mucosal linings, which consequently release stored mediators by granule exocytosis and synthesize leukotrienes and cytokines. The allergic reaction is further promoted by the recruitment of Th2 cells, eosinophils and basophils in the nasal cavity. Th1 cells, into which naive CD4^+^ T cells preferentially differentiate in the presence of IL-12, IL-15, IL-18, IL-2 and interferon (IFN)-γ, play an important role in not only an induction of cell-mediated immunity but also inhibition of Th2 responses [9,10,11]. Above all, IL-12 is essential for polarizing the antigen specific Th1 responses [12,13,14,15]. Therefore, cytokines involved in Th1-biased response are considered to attenuate Th2-mediated allergic responses. Furthermore, clinical studies have demonstrated that reduced IFN-γ secretion by Th1 cells in neonates is associated with the subsequent development of atopy [16,17,18]. 

OK-432, a preparation of a low-virulence strain (Su) of *Streptococcus pyogenes* (Group A) killed by a penicillin and lyophilized, was developed by Okamoto et al. [19], and has often been used as an immunotherapeutic agent in many types of cancer. OK-432 has been reported to activate immunocompetent cells such as macrophages, T cells, and natural killer (NK) cells, and exerts an anti-tumor effect by these cells producing IL-1, IL-2, IL-6, tumor necrosis factor (TNF)-α and IFN-γ [20,21,22,23,24]. In addition, recent studies have suggested that OK-432 induces IL-12 and polarizes the T cell response to Th1 dominant milieu in mice [25,26] However, it remains to be investigated which bacterial component largely contributes to the cytokine induction.

During the last decade, it has been extensively elucidated that TLRs are mammalian homologues of the *Drosophila* Toll receptor and have a role in innate recognition of bacteria [27]. Furthermore, TLR 2 and TLR4 are reported to be implicated in the recognition of various bacterial cell wall components, such as lipopolysaccharide (LPS) [28]. Above all, TLR2 is reported to be an agonist of peptidoglycan as a central structure of OK-432.

In the present study, we examined the role of TLR2 for the recognition for OK-432. Furthermore, we examined the effect of peritoneal injection of OK-432 on murine allergic rhinitis model.

## 2. Materials and Methods

### 2.1. Mice

Six-week-old female C3H/HeN and C3H/HeJ mice were purchased from CLEA Japan, Inc. (Meguro, Tokyo, Japan). C3H/HeJ mice are a non-responder strain to LPS. Six-week-old TLR2 ko mice (C57BL/6 background) were provided by Prof. Akira (Department of Host Defense, Research Institute for Microbial Diseases, Osaka University, Osaka, Japan). Wild-type C57BL/6 mice were used as a control for TLR2 ko mice. These mice were maintained under specific pathogen-free conditions and received an ovalbumin (OVA)-free diet at the laboratory of the animal research center of Shimane University. All mice were 6–7 weeks of age at the beginning of individual experiments. Animal care and experimental procedures were approved by the Animal Research Committee of Shimane University and conducted according to the Regulations for Animal Experimentation at Shimane University.

### 2.2. Reagents and Antibodies

OK-432 was provided by Chugai Pharmaceutical Co., Ltd [19]. LPS from *Escherichia coli* serotype B6:026, anisomycin, phorbol myristate acetate (PMA), dicoumarol, and *p*-nitrophenyl phosphate (pNPP) were obtained from Sigma-Aldrich Co (St Louis, MO, USA). Concentrations of IL-4, IL-12 p40 and IFN-γ in the culture supernatants were measured by ELISA using Quantikine ELISA kits (R&D Systems, Minneapolis, MN, USA) according to the manufacturer’s instructions. For Western blotting, anti-murine monoclonal IL-5 and IL-13 antibodies were purchased from Genzyme (Minneapolis, MA, USA).

### 2.3. Measurement of Cytokine Production by Spleen Cells 

Splenic macrophages from C3H/HeN, C3H/HeJ, C57BL/6 or TLR2 knock-out mice were incubated in RPMI with 10% fetal calf serum (FCS) for 2 h at 37 °C in 96-well tissue culture clusters. Subsequently, the nonadherent cells were removed by gentle aspiration of the cell suspension. The adherent cells were further cultured in complete RPMI with 10% FCS in the presence of Lipid A (1 µg/mL), lipoprotein (1 µg/mL) or OK-432 for 48 h, and supernatants were then harvested for cytokine analysis [29,30].

### 2.4. Measurement of Cytokine Production by Peritoneal Macrophages

C3H/HeN, C3H/HeJ, C57BL/6 or TLR2 ko mice were intraperitoneally (i.p.) injected with 100 µg OK-432, because a sufficient quantity of peritoneal macrophages could be obtained with that amount [31].

Subsequently, peritoneal macrophages, harvested at 12 h post-injection, were incubated in RPMI with 10% FCS for 48 h at 37 °C in 96-well standard tissue culture clusters, and then supernatants were harvested for cytokine analysis [32]. Peritoneal macrophages were prepared as follows. Peritoneal cells were washed and resuspended in RPMI with heat-inactivated 10% FCS and plated with 1 × 10^6^ cells/well in 12-well plates. After incubation for 18 h, the floating cells were washed away and adherent cells were used as peritoneal macrophages.

### 2.5. Immunization Protocol and Treatment

Mice were intraperitoneally immunized with 100 µg OVA absorbed with 100 µg ALUM on days 0 and 7. OK-432 or phosphate-buffered saline (PBS)-treated mice were i.p. injected with OK-432 (100 mg) or PBS on day 0 and 7. Sera and splenic CD3^+^ T cells were obtained at one-week intervals after final immunization. Intraperitoneal immunization was followed by daily challenge with OVA diluted by sterile normal saline intranasally (20 µL of 25 mg/mL OVA per mouse) from day 21 to 28.

### 2.6. Measurement of Cytokine Production by CD3^+^ Splenic T Cells

Splenic cells were incubated on a nylon wool column at 37 °C in 5% CO_2_ for 60 min. T cells (5 × 10^5^) and mitomycin C-treated naive splenocytes (5 × 10^5^) were cultured in 96-well cell culture plates (Falcon, Becton Dickinson, Franklin Lakes, NJ, USA) with 200 µg OVA. After 48 h of culture, the cultured supernatants were collected and the amounts of secreted IL-4 and IFN-γ in the supernatants were determined by an ELISA. For cellular proliferation, the cultures were pulsed with 1 µCi/well of tritiated thymidine ([3H]TdR) for an additional 6 h after 48 h of culture. Incorporation of [3H]TdR was determined by liquid scintillation counting [29,30].

### 2.7. Measurement of OVA-Specific IgE, IgG_1_ and IgG_2a_

Levels of OVA-specific IgE, IgG_1_ and IgG_2a_ were determined by an ELISA. Sample wells of an ELISA plate were coated with OVA overnight and then blocked with 1% bovine serum albumin (BSA) in borate-buffered saline (0.05 M borate, 0.15 M NaCl, pH 8.6, 100 µL/well) at 37 °C for 30 min. Diluted samples (100 µL/well) were incubated for 90 min at room temperature (samples for IgE, IgG_1_ and IgG_2a_ were diluted 1:100, 1:1000 and 1:5, respectively). The plates were washed with borate-buffered saline with 0.05% Tween 20 and incubated with peroxidase-conjugated anti-mouse IgE (Nordic Immunology, Minneapolis, MN, USA), IgG_1_ or IgG_2a_ (Zymed Laboratories, Inc, San Francisco, CA, USA) for 90 min at room temperature. After further washing, plates were incubated for 20 min at room temperature with 100 µL/well of o-phenylendiamine solution (1µg/mL with 3% H_2_O_2_), and optical density (OD) was read at 492 nm [29,30].

### 2.8. Evaluation of Nasal Symptom

Before the final intranasal challenge with OVA or PBS, the mice were placed into an observation cage (one animal/cage) for about 10 min for acclimatization. After the intranasal challenge with OVA or PBS, the mice were placed into the observation cage again and the number of sneezes was counted for 10 min by the method of Sugimoto et al. [33].

### 2.9. Histological Examination

Mice were killed 12 h after the last intranasal (i.n.) challenge with OVA or PBS. The heads were removed and fixed in 10% formaldehyde solution for 24 h at room temperature. After fixation, the heads were decalcificated in 5% formic acid for 36 h at room temperature and neutralized in 5% sodium sulfate solution for 12 h at room temperature. Coronal nasal sections were then stained with hematoxylin and eosin, and the number of eosinophils in each side of the posterior edge of the nasal septum was counted microscopically using Image Pro Plus Imaging software version 4.0.011 (Media Cybernetics, Del Mar, CA, USA).

### 2.10. Western Blot Analysis

Proteins were obtained from the nasal mucosa of each mouse 12 h after the final nasal challenge by using lysis buffer (50 mM hydroxyethyl piperazineethanesulfonic acid (HEPES), pH 7.0, 150 mM NaCl, 10% glycerol, 1% Triton X-100, 1.5 mM MgCl_2_, 1 mM ethylene glycol tetraacetic acid (EGTA), 100 mM NaF, 10 mM NaPP_i_, 1 mM Na_3_VO_4_, 1 mM phenylmethanesulfonyl fluoride with aprotinin and leupeptin at 10 µg/mL). Samples were centrifuged (15,000 rpm, 5 min), and the supernatants were stored at −80 °C for further experiments. Lysates were analyzed on a 15% sodium dodecyl sulfate (SDS)–polyacrylamide gel, and the proteins were transferred to Immobilon polyvinylidene difluoride membranes (Millipore, Bedford, MA, USA). Membranes were blocked with 1% bovine serum albumin in Tris-buffered saline containing 0.05% Tween-20 (TBST) for 1 h, and western blot analysis was performed followed by detection using enhanced chemiluminescence system (Amersham Pharmacia Biotech, Amersham, UK) according to the manufacturer’s instructions.

### 2.11. Statistical Analysis

The statistical significance of the data was determined by Student’s *t*-test. A value of *p* < 0.05 was considered as significant.

## 3. Results

### 3.1. Effect of OK-432 on IL-12 Production by Splenic Macrophages Derived from C3H/HeN, C3H/HeJ, TLR2 KO and C57/BL6 Mice In Vitro

**Figure 1:**
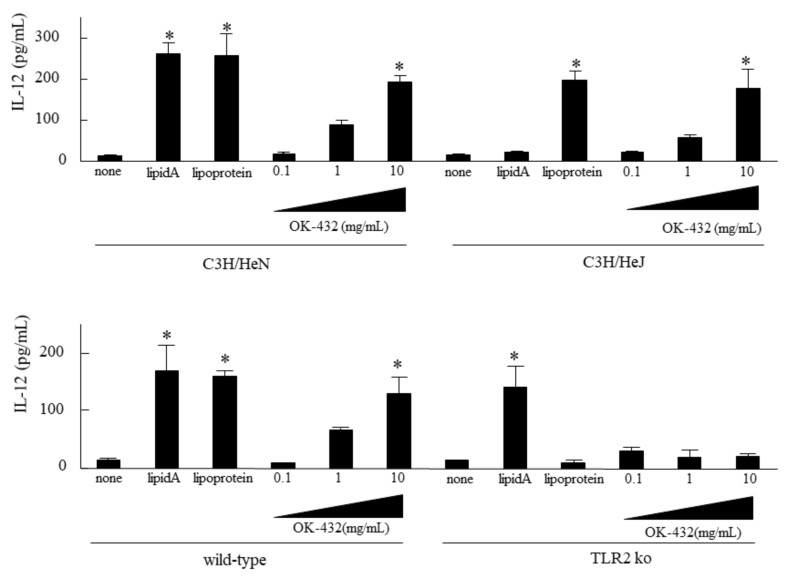
IL-12 production by splenic macrophages from C3H/HeN, C3H/HeJ, wild-type or TLR2 ko mice in response to OK-432. The macrophages from the spleen of C3H/HeN, C3H/HeJ, wild-type or TLR2 knock-out mice were cultured in the presence of lipid A, lipoprotein or OK-432 for 48 h at 37 °C. IL -12 production by macrophages was measured by an ELISA. The data are representative of four independent experiments using pooled cells from five mice and are shown as the mean of triplicate determinations ± standard deviation (SD). *, *p* < 0.005.

To examine which type of TLRs recognized OK-432 in mice, IL-12 production by splenic cells derived from C3H/HeN mice was assessed in vitro, in comparison with those from C3H/HeJ, C57/BL6 or TLR2 knock-out mice. IL-12 production was significantly increased in the splenic cells stimulated by OK-432 derived from C3H/HeN mice as well as C3H/HeJ mice (Figure 1). Moreover, IL-12 production was higher in the splenic cells derived from C57BL/6 mice stimulated by OK-432 than those derived from TLR2 knock-out mice.

### 3.2. Effect of OK-432 on IL-12 Production by Murine Peritoneal Macrophages In Vivo

**Figure 2:**
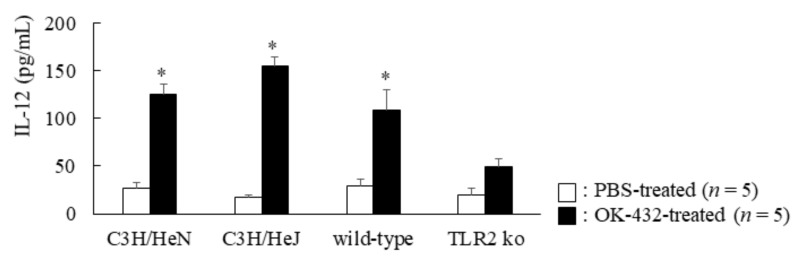
IL-12 production by peritoneal macrophages from intraperitoneally PBS or OK-432-treated mice. The peritoneal macrophages from phosphate-buffered saline (PBS)-treated (white bars) or OK-432-treated (black bars) mice sensitized with OVA were cultured in the presence of OVA for 48 h at 37°C. IL-12 production by murine peritoneal macrophages were measured by an ELISA. Data were obtained from three independent experiments and are expressed as the mean of triplicate determinations ± SD. Per experimental group, five mice were used. *, *p* < 0.005.

Next, we examined the in vivo effect of OK-432. C3H/HeN, C3H/HeJ, TLR2 knock-out mice or wild-type mice were i.p. injected with 100 µg OK-432. Subsequently, peritoneal macrophages, harvested at 12 h post-injection, were incubated in RPMI with 10% FCS for 48 h at 37 °C in 96-well standard tissue culture clusters, and then supernatants were harvested for cytokine analysis. As shown in Figure 2, IL-12 production was higher in the peritoneal macrophages derived from wild-type mice than TLR2 knock-out mice. However, there was no difference in IL-12 production by macrophages between C3H/HeN and HeJ mice.

### 3.3. The Effect of OK-432 for Th2 Responses and Allergic Rhinitis in C3H/HeN and C3H/HeJ Mice Sensitized with OVA at Induction Phase

**Figure 3:**
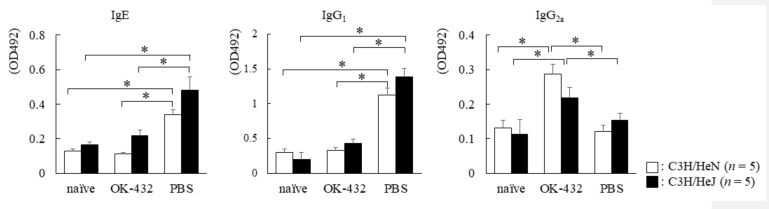
Serum levels of OVA-specific Igs in C3H/HeN (white bars) or C3H/HeJ (black bars) mice sensitized with OVA. Individual levels of OVA-specific Igs were determined by an ELISA in C3H/HeN or C3H/HeJ mice on day 14 after i.p. injections with OVA/ALUM on day 0 and day 7, or naive C3H/HeN or C3H/HeJ mice without sensitization of OVA. PBS-treated mice were intraperitoneal (i.p.) injected with PBS on day 0 and day 7. OK-432-treated mice were i.p. injected with 100 mg OK-432 on day 0 and day 7. Data were obtained from three independent experiments and are expressed as the mean of triplicate determinations ± SD. Per experimental group, five mice were used. *, *p* < 0.005.

To determine which type of T cell responses preferentially developed in C3H/HeN and C3H/HeJ mice treated with OK-432 at induction phase by OVA sensitization, we examined OVA-specific IgE/IgG_1_/IgG_2a_ production in sera on day 14 after the first immunization with OVA/ALUM. PBS-treated C3H/HeN and C3H/HeJ mice had higher OVA-specific IgE and IgG_1_ levels in sera than those of OK-432-treated mice after OVA sensitization (Figure 3). On the other hand, PBS-treated C3H/HeN and C3H/HeJ mice had lower OVA-specific IgG_2a_ levels in sera than those of OK-432-treated mice after OVA sensitization. Therefore, we separated T cells from the spleen of mice treated with OK-432 or PBS at the induction phase.

**Figure 4:**
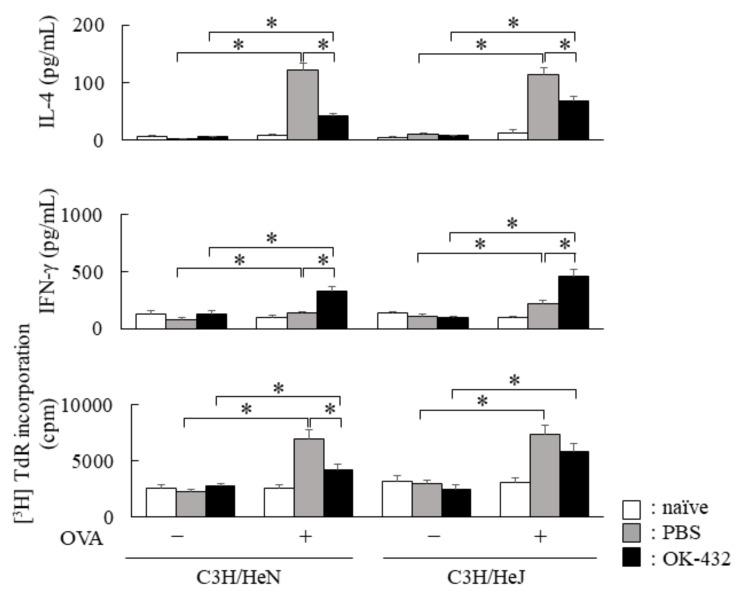
Cytokine production by spleen T cells from OK-432-treated or PBS-treated mice at the induction phase. The enriched T cells (5 × 10^5^ cells) from the spleen of OK-432-treated (black bars) or PBS-treated (hatched bars) mice sensitized with OVA or naive mice (white bars) were cultured with OVA in the presence of MMC-treated spleen cells (5 × 10^5^ cells) for 48 h at 37 °C. All mice were immunized with OVA/ALUM. The proliferative responses of splenic T cells were measured by an incorporation of [^3^H]TdR, and data was represented by CPM (counts per minute). IL-4 and interferon (IFN)-γ production by T cells was measured by an ELISA. The data are representative of four independent experiments using pooled cells from five mice and are shown as the mean of triplicate determinations ± SD. *, *p* < 0.005.

In response to OVA, splenic CD3^+^ T cells of PBS-treated C3H/HeN and C3H/HeJ mice sensitized with OVA actually produced IL-4, whereas IL-4 production significantly decreased in OK-432-treated C3H/HeN and C3H/HeJ mice sensitized with OVA (Figure 4). Furthermore, IFN-γ production in response to OVA increased in the culture supernatants of splenic T cells from OK-432-treated C3H/HeN and C3H/HeJ mice sensitized with OVA.

**Figure 5:**
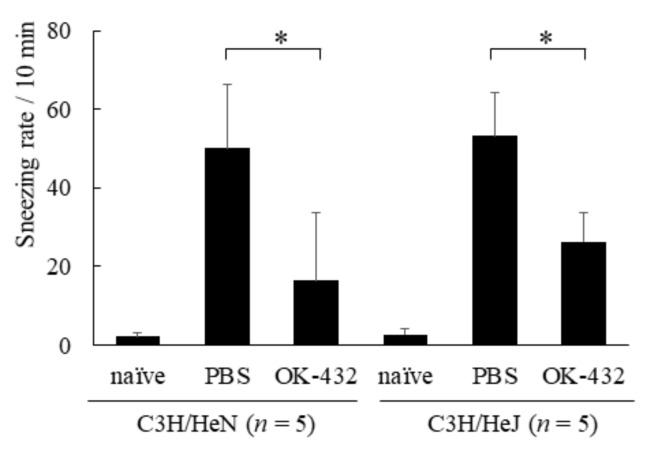
Sneezing rate in a murine model of C3H/HeN or C3H/HeJ mice systemically sensitized with OVA and receiving subsequent intranasal challenge of OVA. Naive mice underwent no systemic sensitization. PBS-treated mice were i.p. injected with PBS on day 0 and day 7. OK-432-treated mice were i.p. injected with 100 mg OK-432 on day 0 and day 7. Data were obtained and expressed as the mean of triplicate determinations ± SD. Per experimental group, five mice were used. *, *p* < 0.005.

**Figure 6:**
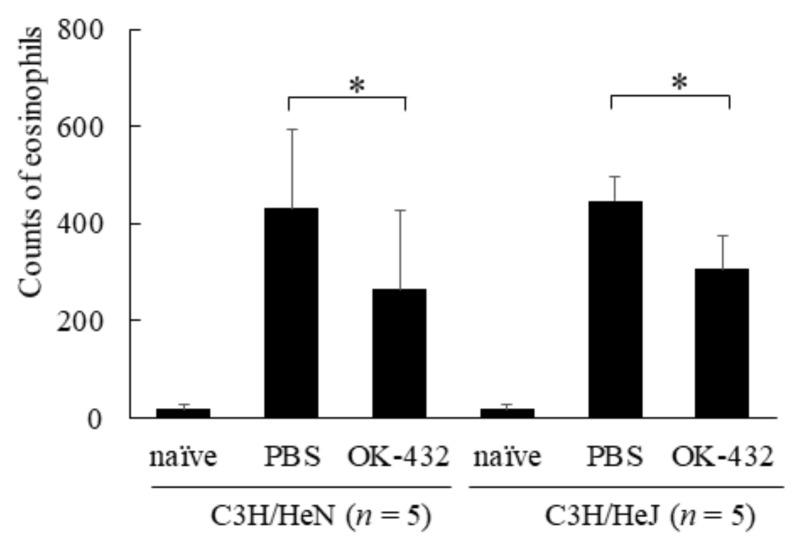
Eosinophil infiltration in nasal mucosae in a murine model of C3H/HeN or C3H/HeJ mice systemically sensitized with OVA and receiving subsequent intranasal challenge of OVA. Naive mice underwent no systemic sensitization. PBS-treated mice were i.p. injected with PBS on day 0 and day 7. OK-432-treated mice were i.p. injected with 100 mg OK-432 on day 0 and day 7. Data were obtained and expressed as the mean of triplicate determinations ± SD. Per experimental group, five mice were used. *, *p* < 0.005.

**Figure 7:**
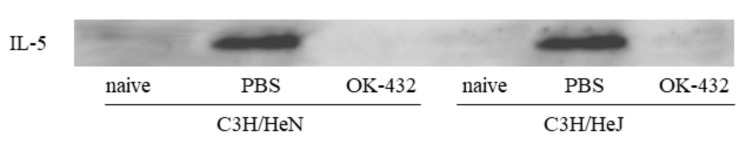
IL-5 expressions of nasal mucosae assessed by Western blot assay, in a murine model of C3H/HeN or C3H/HeJ mice systemically sensitized with OVA and receiving subsequent intranasal challenge of OVA. Naive mice underwent no systemic sensitization. PBS-treated mice were i.p. injected with PBS on day 0 and day 7. OK-432-treated mice were i.p. injected with 100 mg OK-432 on day 0 and day 7. IL-5 expressions were detected in PBS-treated C3H/HeN and C^3H^/HeJ mice, but not found in OK-432-treated-mice of both strains.

In addition, we examined the effects of OK-432 on nasal symptoms and histopathology in a murine model of allergic rhinitis. The number of sneezes within a 5 min period after the final intranasal challenge with OVA or PBS was carefully counted in mice. There were differences in sneezing rates, eosinophilic infiltrations and IL-5 expressions in nasal mucosae between PBS-treated and OK-432-treated mice of both C3H/HeN and C3H/HeJ strains (Figure 5, Figure 6 and Figure 7).

### 3.4. The Effect of OK-432 for Th2 Responses and Allergic Rhinitis in C57/BL6 and TLR2 Knock-Out Mice Sensitized with OVA at Induction Phase

**Figure 8:**
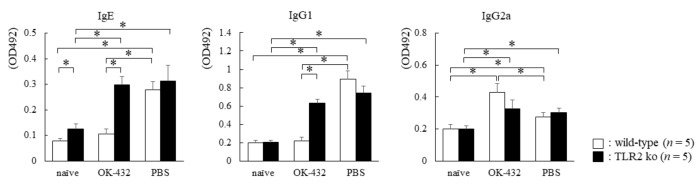
Serum levels of OVA-specific Igs in wild-type (white bars) or TLR2 knock-out (black bars) mice sensitized with OVA. Individual levels of OVA-specific Igs were determined by an ELISA in wild-type or TLR2 knock-out mice on day 14 after i.p. injections with OVA/ALUM on day 0 and day 7, or naive wild-type or TLR2 knock-out mice without sensitization of OVA. PBS-treated mice were i.p. injected with PBS on day 0 and day 7. OK-432-treated mice were i.p. injected with 100 mg OK-432 on day 0 and day 7. Data were obtained from three independent experiments and are expressed as the means of triplicate determinations ± SD. Per experimental group, five mice were used. *, *p* < 0.005.

We also examined the effect of OK-432 on Th2 responses and allergic rhinitis in TLR2 knock-out mice in comparison with wild-type of C57BL/6 mice. As shown in Figure 8, PBS-treated wild-type mice had higher OVA-specific IgE and IgG_1_ levels in sera than those of OK-432-treated wild-type mice after OVA sensitization. PBS-treated wild-type mice had lower OVA-specific IgG_2a_ levels in sera than those of OK-432-treated wild-type mice after OVA sensitization.

On the other hand, OVA-specific IgE and IgG_1_ levels in sera derived from OK-432-treated TLR2 knock-out mice did not significantly decrease in comparison with those of PBS-treated TLR2 knock out mice. There was no difference in the level of OVA-specific IgG_2a_ between the OK-432-treated and the non-treated TLR2 knock-out mice sensitized with OVA.

**Figure 9:**
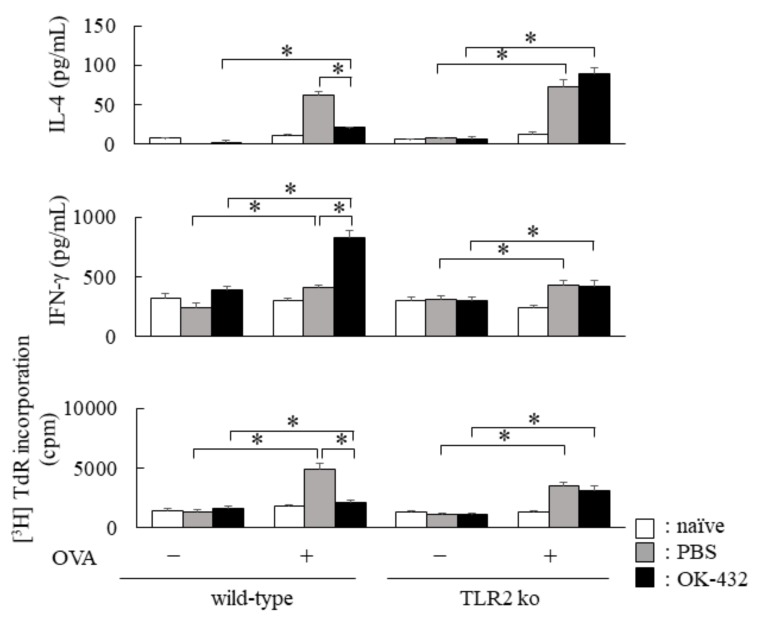
Cytokine production by splenic T cells from OK-432-treated or PBS-treated mice at the induction phase. The enriched T cells (5 × 105 cells) from the spleen of OK-432-treated (black bars) or PBS-treated (hatched bars) mice sensitized with OVA or naive mice (white bars) were cultured with OVA in the presence of MMC-treated spleen cells (5 × 10^5^ cells) for 48 h at 37 °C. All mice were immunized with OVA/ALUM. The proliferative responses of splenic T cells was measured by an incorporation of [^3^H]TdR. IL-4 and IFN-γ production by the splenic T cells was measured by an ELISA. The data are representative of four independent experiments using pooled cells from five mice and are shown as mean of triplicate determinations ± SD. *, *p* < 0.005.

Furthermore, we examined the cytokine production by splenic T cells derived from C57BL/6 and TLR2 knock-out mice treated with OK-432 or PBS at the induction phase. Splenic CD3^+^ T cells of PBS-treated C57BL/6 mice sensitized with OVA produced IL-4 in response to OVA, whereas IL-4 production was significantly reduced in OK-432-treated C57BL/6 mice sensitized with OVA (Figure 9).

**Figure 10:**
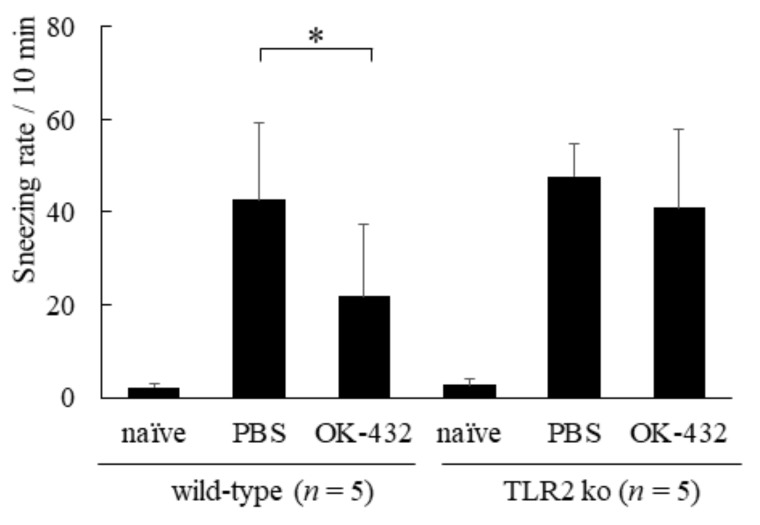
Sneezing rate in a murine model of wild-type and TLR2 knock-out mice systemically sensitized with OVA and receiving subsequent intranasal challenge of OVA. Naive mice underwent no systemic sensitization. PBS-treated mice were i.p. injected with PBS on day 0 and day 7. OK-432-treated mice were i.p. injected with 100 mg OK-432 on day 0 and day 7. Data were obtained and expressed as the mean of triplicate determinations ± SD. Per experimental group, five mice were used. *, *p* < 0.005.

The IFN-γ production in response to OVA increased in the culture supernatant of splenic T cells from OK-432-treated C57/BL6 mice sensitized with OVA. On the other hand, there were no differences in IL-4 and IFN-γ production by splenic T cells between OK-432-treated and PBS-treated TLR2 knock-out mice. The number of sneezes during a 5 min period following the last i.n. challenge with OVA or PBS was carefully counted in mice. The number of sneezes was significantly higher in PBS-treated wild-type mice than in OK-432-treated wild-type mice (Figure 10).

**Figure 11:**
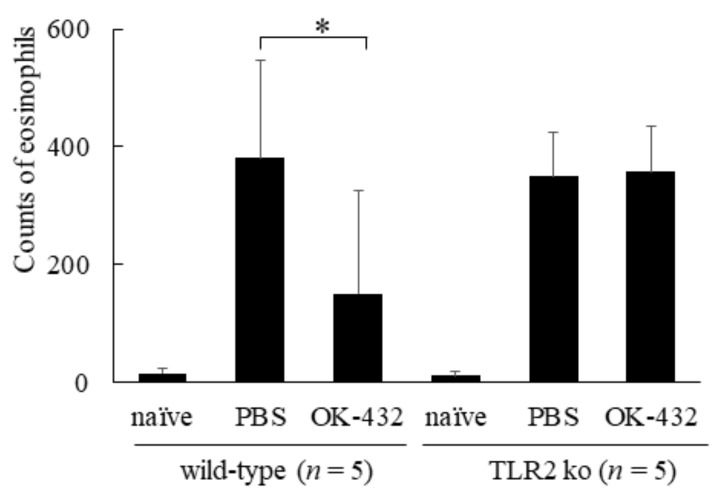
Eosinophil infiltration in nasal mucosae in a murine model of wild-type and TLR2 knock-out mice systemically sensitized with OVA and receiving subsequent intranasal challenge of OVA. Naive mice underwent no systemic sensitization. PBS-treated mice were i.p. injected with PBS on day 0 and day 7. OK-432-treated mice were i.p. injected with 100 mg OK-432 on day 0 and day 7. Data were obtained and expressed as the mean of triplicate determinations ± SD. Per experimental group, five mice were used. *, *p* < 0.005.

There was no significant difference in the number of sneezes between PBS-treated and OK-432-treated TLR2 knock-out mice. To elucidate the effect of OK-432 at the late phase response of the eliciting phase, we counted the number of eosinophils infiltrating into the nasal mucosa 12 h after the last i.n. challenge with OVA or PBS. The infiltration of eosinophils into the nasal mucosa aggravated in PBS-treated wild-type mice after i.n. challenge with OVA was significantly higher in comparison with that of OK-432 -treated wild type mice (Figure 11).

**Figure 12:**
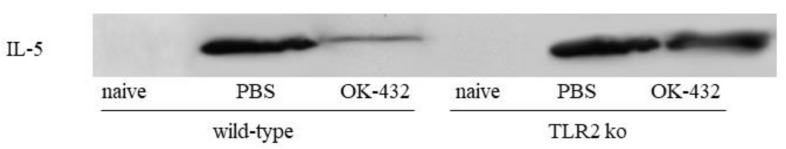
IL-5 expressions of nasal mucosae assessed by western blot assay, in a murine model of wild-type and TLR2 knock-out mice systemically sensitized with OVA and receiving subsequent intranasal challenge of OVA. Naive mice underwent no systemic sensitization. PBS-treated mice were i.p. injected with PBS on day 0 and day 7. OK-432-treated mice were i.p. injected with 100 mg OK-432 on day 0 and day 7. IL-5 expression was detected in PBS-treated wild type of mice and TLR 2 knock-out mice. Interestingly, it was also detected in OK-432-treated TLR2 knock-out mice, whereas it was downregulated in PBS-treated wild-type mice.

On the contrary, the number of eosinophils infiltrating into nasal mucosa was not reduced even after OK-432 treatment in TLR2 knock out mice. We also dissected nasal mucosa and analyzed Th2-type cytokine expression by a western blot assay. The expression of IL-5 was significantly inhibited by OK-432 treatment in wild-type mice (Figure 12).

However, no difference was found in IL-5 expression between OK-432 treatment and PBS treatment in TLR2 knock-out mice after OVA inhalation. We were unable to detect IL-4 by a Western blot assay.

## 4. Discussion

In this study, we extensively investigated the efficacy of OK-432 treatment in the Th2 responses via TLR2 focused on cell signaling of macrophages. Up to now, anti-tumor effects of OK-432 were indicated by many investigations, even though the exact recognition mechanism of OK-432 by macrophages remained to be investigated.

OK-432 is a preparation of a low-virulence strain (Su) of *Streptococcus pyogenes* (Group A) killed by a penicillin and lyophilized [19]. OK-432 contains lipoteichoic acid, peptidoglycan and M-protein [34]. Peptidoglycan is a central structure of OK-432 and reported to be an agonist of TLR2 [28]. TLR2 is constitutively expressed on macrophages and various cytokines such as IL-12, IL-15 and TNF-α, are produced by macrophages via TLR2 stimulation. Above all, IL-12 is essential for polarizing the antigen specific Th1 responses [12,13,14,15,35]. Upregulation of Th1 responses has been considered to inhibit Th2 responses and atopic diseases [36,37,38,39,40]. Therefore, we focused on the role of OK-432 for the -production of IL-12 of macrophages via TLR2 stimulation.

First of all, we established the exact means of production of IL-12 by murine macrophages stimulated with OK-432. OK-432 enhanced IL-12 release from splenic macrophages derived from naive C57BL/6 strain of mice. However, no effects were observed in macrophages derived from TLR2 knock-out mice, even after adding OK-432. In contrast, splenic macrophages derived from C3H/HeJ mice, as TLR4 gene mutant mice, significantly produced IL-12 via stimulation with OK-432, similarly to splenic macrophages derived from C3H/HeN mice. Therefore, these data indicate that OK-432 may act via the TLR2 pathway.

Second, we examined the in vivo role of OK-432 on the IL-12 production of macrophages. With an intraperitoneal injection of OK-432 into C57/BL6 mice, peritoneal macrophages of this strain of mice produced a significant amount of IL-12, in comparison of those of TLR2 knock-out mice. However, there was no significant difference in IL-12 production of peritoneal macrophages between C3H/HeN or C3H/HeJ mice i.p. injected with OK-432. Based on these in vivo data, we can be sure that TLR2, but not TLR4, plays an essential role in the production of IL-12 by macrophages stimulated with OK-432.

Then, we analyzed immunopharmacological effects of OK-432 on nasal symptoms and histopathology in a murine model of allergic rhinitis. When i.p. injection with OK-432 to C3H/HeN, C3H/HeJ and C57 BL/6 mice was begun immediately after OVA sensitization, Th2 responses significantly reduced. Moreover, allergic inflammation in nasal mucosa was also induced in these mice i.p. injected with OK-432. In contrast, i.p. injection with OK-432 to TLR2 knock-out mice did not influence Th2 responses and nasal allergic inflammation. Up-regulation of IL-12 production by macrophages stimulated with OK-432 via TLR2 in vivo seemed to successfully inhibit the Th2 augmentation at induction phase of murine allergic rhinitis model.

Allergic rhinitis is a complex phenomenon driven predominantly by Th2-type cells [1,2,3,4,5]. Allergic rhinitis is characterized by the overproduction of Th2 cytokines, which initiate and sustain the allergic asthmatic inflammatory responses by enhancing the production of IgE and the growth [6], differentiation, and recruitment of mast cells, basophils, and eosinophils. Th2 response is inhibited by IFN-γ-producing Th1 cells. Clinical studies have demonstrated that reduced IFN-γ secretion in neonates is associated with the subsequent development of atopy [16,17,18]. Furthermore, a predisposition toward the overproduction of Th1 cytokines may protect against atopy, because patients with multiple sclerosis [41], rheumatoid arthritis [42] or tuberculosis [43], conditions associated with increased production of Th1 cytokines, have a reduced predisposition toward the development of atopy. According to the hygiene hypothesis [44], bacterial infection inhibits Th2 polarization. It seems to be very important to establish the strategy to enhance Th1 response in neonate clinically. From our data, OK-432 induces IL-12 production from macrophages via TLR2 and inhibits Th2 responses and atopic inflammation, including allergic rhinitis. Administration of OK-432 into neonates may, therefore, be a useful strategy for inhibition of Th2 responses and allergic inflammation. Although OK-432 is a whole bacterial component, the specific part of OK-432 which affects TLR family protein as an agonist is still unknown. However, intracellular signal transduction pathway of the activated human monocytes with OK-432 has been clearly demonstrated by Olsnes et al. [45,46]. Hence, we will examine which part of OK-432 affects TLR family protein as an agonist in future. Moreover, macrophage/dendritic cell-derived cytokines, such as IL-12, IL-15 and IL-18, are at least partly responsible for early IFN-γ production from NK and γδT cells, and consequently Th1 cell differentiation. Alternatively, OK-432 may contain ligands for NK and γδT cells and directly stimulate produced IFN-γ.

OK-432 is clinically administered intramuscularly, subcutaneously, intradermally or directly to a tumor as a treatment for malignant tumors, intrathoracically for carcinomatous pleural effusion, intraperitoneally for carcinomatous ascites, and intracystically for lymphangioma. A feasible method of administration for allergic patients would be intramuscular, subcutaneous, or intradermal administration, the same method as used for malignant tumor patients. Side effects of OK-432 for malignant tumors have been reported, such as erythema, discomfort at the injection site, swelling and pyrexia according to package insert of OK-432, but these side effects improve within a week.

In summary, intraperitoneal injection with OK-432 into mice reduced Th2 responses in the induction phase of allergic responses via IL-12 production by macrophages. OK-432 was considered to affect as an agonist of TLR2 on macrophages. These results thus offer a new approach using OK-432 for the prophylactic and therapeutic treatment of allergic disorders, such as allergic rhinitis.
